# The Origin and Impact of Ideals in eHealth Research: Experiences From the U-CARE Research Environment

**DOI:** 10.2196/resprot.3202

**Published:** 2014-05-23

**Authors:** Jonas Sjöström, Louise von Essen, Helena Grönqvist

**Affiliations:** ^1^U-CAREDepartment of Public Health and Caring SciencesUppsala UniversityUppsalaSweden; ^2^Information SystemsDepartment of Informatics and MediaUppsala UniversityUppsalaSweden

**Keywords:** research management, stakeholders, innovation, accountability, rigor, relevance, sustainability

## Abstract

**Background:**

The prevalence of information technology (IT) in society is a foundation for new modes of interaction between patients and health specialists. IT plays an important role in the renewal of care. Several countries have incorporated eHealth plans into their national health strategies. Part of the eHealth evolution concerns Internet psychological treatment and psychosocial care. These interventions are complex to design and evaluate due to legal, ethical, organizational, technical, and methodological challenges.

**Objective:**

The objective of our study was to seek to make explicit contributions to the understanding of ideals in eHealth research, and illuminate their implications for establishing an effective research environment. Our analysis draws from three years of experience in establishing an eHealth research environment, and the literature.

**Methods:**

We worked inductively to characterize challenging research ideals, and their origins, in our environment. Thereafter, we made a selective search of the literature to scrutinize and illuminate each ideal and it’s implications.

**Results:**

In this work, we propose a structured approach to address ideals in eHealth research. The scrutinized ideals are accountability, innovation, rigor, relevance, and sustainability. The approach supports researchers to systematically understand the ideals, their origin, and to manage their implications within an eHealth research environment.

**Conclusions:**

The complexity of eHealth research causes a need for sustainable, multi-disciplinary research environments. There is a need for a structured approach to organize eHealth research. The proposed approach helps to systematically scrutinize ideals, thus promoting high quality research.

## Introduction

### The Prevalence of Information Technology

The prevalence of information technology (IT) in society is a foundation for new modes of interaction between patients and health specialists. While the field of eHealth is still in its infancy, it is clearly conceived of as an important strategy for the future of health care. This is signified by the status of eHealth as a key area in the Digital Agenda for Europe and the Innovation Union, both major parts of the Europe 2020 strategy presented by the European Commission in 2010.

Part of the eHealth evolution is the growth of Web-based psychological treatment and psychosocial care. Web-based self help is effective for psychiatric disorders and promotion of health behaviors [1,2]. The approach is also promising with regard to costs, by using less therapist time per effectively treated patient compared to face-to-face therapy [3,4].

### eHealth Research Challenges

Web-based interventions are complex to develop and evaluate, and substantial investments are required [5]. Several authors have addressed the challenges related to eHealth research [1,6-8]. Barak et al [1] highlight the challenges related to: (1) transition from face-to-face to Web-based communication, (2) ethical issues related to patient confidentiality and handling of emergency situations, (3) laws and regulations, and (4) practical and technical concerns that follow from appropriating technology for critical activities in organizations. Whitehouse et al [7] discussed legal, ethical, and governance challenges, and Ahern [6] pointed out both conceptual and methodological challenges, as well as and design issues, concluding that there is a need for more research in order to “leverage the opportunities for public health impact afforded by eHealth programs”.

The challenges with eHealth research at least partly originate from the environment in which the research takes place. Researchers need to relate to ideals put forward by numerous stakeholders including, but not limited to, the research community, legislative bodies, and the media. Ideals are in flux, continually reinforced and challenged by stakeholder groups [9]. In this work, we propose an approach to systematically address ideals in eHealth research. The approach supports researchers to understand the ideals and their origins, and to adapt their work to comply with the ideals. We seek to contribute to the understanding of stakeholder ideals, and their implications for establishing an effective research environment. Our analysis draws from three years of experience of establishing an eHealth research environment and the literature.

## Methods

### The Research Setting

The research setting at hand is a multi-disciplinary research environment at Uppsala University, Uppsala, Sweden, between 2010 and 2014, and the development of the Uppsala University Psychosocial Care (U-CARE) program. The program was established to support three randomized controlled trials on Internet-based treatment of depression and anxiety for patients with somatic disease. The development may be characterized as an entangled design of the research environment, trials, interventions, and software. The U-CARE program involves academics from psychology, medicine, information systems, caring sciences, and economics, as well as health practitioners. A set of organizational decisions was made at the inception of the program. A scientific advisory board was established, including a set of scholars with expertise in their respective academic fields. The scientific advisory board meets with U-CARE staff annually to provide feedback on the U-CARE research activities. In addition, coordination groups, including researchers from different disciplines, were established to coordinate the work between and within various studies. Information systems researchers were contributing practically with software development, as well as doing research on information systems issues.

### eHealth Services for Patients

U-CARE is oriented toward eHealth services for patients. The overall aim of the U-CARE program is to prevent and reduce emotional distress in persons struck by a somatic disease. Being struck by a potentially life threatening disease such as cancer can cause, for example, depression and anxiety [10]. This distress may not only cause human suffering, but can also negatively impact the treatment of the somatic disease and bring about other issues for the individual and society. For example, a depressive state may cause a patient to engage in less physical activity [11], contribute to sleeping problems, and nonadherence to prescribed medications [12]. The interventions evaluated within the trials are based on cognitive behavioral therapy [13,14]. Some of the interventions include psychosocial care consisting of information and interactive support. In the interactive parts, patients become part of an online community, allowing them to interact with peers in discussion forums, online chats, and through internal messages. Patients are recruited to trials at various hospitals in Sweden through collaboration with hospital staff.

### The eHealth Software

The eHealth software at hand was designed to be configurable in a number of ways to facilitate the diverging needs within the three initial trials. An example of this flexibility is the option to either compose new interventions that are to be evaluated in additional trials, these possibly based on the intervention content in the original trials, or to reuse the original content. In the same manner, it is possible to use the questionnaires in the original trials, or to use new ones as well as to use the original inclusion and randomization logic, or to develop a new logic for inclusion and randomization. The software and the interventions have already attracted a number of research groups who will perform observational or intervention studies via the software. At this point, nine research groups are in different stages of planning and starting studies. These studies are both benefitting from and contributing to knowledge and technology within the U-CARE environment. In addition, the ethical approval required for each trial increased the experience of ethical considerations in online trials among U-CARE staff.

### The Research Approach

Our research approach follows the pragmatist assumption that design and intervention in a real world setting are effective ways to understand social mechanisms [15,16]. The underlying idea is that social phenomena are more likely to be disclosed in action rather than via observations or interviews. Thus, the development of the U-CARE program and the subsequent interpretation of experiences are based on the notion that design and change in a specific domain is a viable approach to understand the domain [17].

The domain at hand is the evolution of a research environment. Given the complexity of the environment, aiming at researching psychosocial support for people with somatic disease, through collaboration across disciplines and including multiple hospitals, we conceive of the establishment of the research environment as a rich source for reflections on performing eHealth research. During the evolution of the U-CARE environment, a number of situations occurred that were not easily remedied. The situations typically included design complexities that were not anticipated, for example, the uncertainty on how to manage details of IT-reliant communication between patients and staff. Over time, the awareness emerged that these challenges needed to be more systematically addressed to avoid “bottlenecks” in the design process. The experiences connected to these situations were interpreted in the light of relevant literature and abstracted into the results presented in this paper, consisting of five identified ideals that affect eHealth research, and an approach to scrutinizing such ideals. The process was initially inductive, for example, it was based on the prevalent challenges that were encountered during the design of research protocols, patient treatment, and software. Through literature studies, the approach was iteratively refined in an interpretive and hermeneutic process. The resulting approach is thus an abstraction from a single case study [18], for example, the evolution of the U-CARE research environment. The literature, however, indicates that the results are valid in a broader context.

### An Approach to Scrutinize Ideals in eHealth Research

In this section, we provide a structure to scrutinize ideals based on: (1) a practice perspective on organizations, and (2) a stakeholder centric approach. Since the objective of this paper is to discuss the challenges in building an eHealth research environment, there is a need for reflection about the definition of a “research environment”. Numerous theories can be used to explain and analyze organizations. A contemporary view is that organizations may be studied using a “practice lens”. Social science researchers have elaborated on the concept of practice for a long time, and there is no unified view of what it means. We here subscribe to Schatzki’s view on practice [19], recognizing the materiality of the social world, for example, that artifacts affect human action and vice versa. The practice stance, drawing on Giddens’ structuration theory [20], highlights the reciprocal shaping of action and structure. The reciprocity means that social structures govern human behaviors, while at the same time; individuals’ actions reinforce and challenge social structure. The practice view resonates well with the phenomena under scrutiny in this paper; the way that social structures (in this case ideals imposed on research by various external stakeholders) enable and constrain action, for example, they cause challenges in organizing a research environment.

A research environment operates in a context continually influenced by and adapting to external parties, such as academic journals, ethical approval boards, funding agencies, and legislative bodies. An implication of adopting the practice view on the research environment is that a successful research environment needs to adapt to their stakeholders, who define the preconditions for research practice, and evaluate its outcome.

**Table 1:** A structure to scrutinize ideals in eHealth research.

Aspect	Explanatory question
Theoretical discussion and definition	What is the meaning of the ideal and why is it important?
Stakeholders	Who reinforces the ideal?
Impact on the U-CARE environment	How, when, and where did the ideal affect the environment?
Managerial implications	What are the managerial implications for the research environment?

### What, Who, Why, How, When, and Where

An approach to make sense of qualitative data is to appropriate the six interrogatives: (1) what, (2) who, (3) why, (4) how, (5) when, and (6) where [21]. We adapt the interrogatives to the current context to scrutinize each identified ideal, as outlined in Table . In addition, each ideal is discussed from the point-of-view of managerial implications. These include: (1) accountability, (2) innovation, (3) relevance, (4) rigor, and (5) sustainability.

In the remainder of this section, we adopt the approach to scrutinizing ideals to provide an account of each ideal and its impact on the research environment.

### Accountability

#### Theoretical Discussion and Definition

Accountability is a core concern in health care. The meaning of information accountability is that “[...] use of information should be transparent so it is possible to determine whether a particular use is appropriate under a given set of rules, and that the system enables individuals and institutions to be held accountable for misuse” [22]. Accountability in an eHealth context, for example, concerns about privacy issues and avoiding misuse of patient information. As stated in the Universal Declaration of Human Rights [23] (Article 12), “...no one shall be subjected to arbitrary interference with his privacy, family, home, or correspondence, nor to attacks upon his honor and reputation”. A main concern in eHealth is that privacy should be protected at all times. Information access should always be motivated by caregiving needs, and research should be based on informed consent [24]. Accountability is reached when it can be reconstructed how an undesired situation occurred. Accountability is not only a matter of securing safe access to information, but also about making people aware about policies and facilitating transparency in information use [22]. Managing accountability is a challenge both from a knowledge point of view and from a technological point of view. Researchers need to be aware of and comply with detailed legislation and ethics concerning how patient information should be retrieved and handled. Technology needs to be aligned with state-of-the-art practices for security, authentication, and procedures to scrutinize information use and misuse.

#### Stakeholders

Accountability builds on human rights and legislation, and differs between nations. Researchers need to account for the way they manage patient information to research funders, ethical approval boards, journals, and government agencies, and not least the citizens.

#### Managerial Implications

Accountability needs to be addressed using multiple competencies, including law, health, and information technology expertise. While substantial resources are required to find solutions to accountability issues, it is imperative to systematically reuse knowledge and technology to efficiently set up and execute new projects within the eHealth area.

### Innovation

#### Theoretical Discussion and Definition

We adhere to the view of innovation as “...the multi-stage process whereby organizations transform ideas into new/improved products, services or processes, in order to advance, compete, and differentiate themselves successfully in their marketplace” [25]. In health care research, innovation concerns the translation of evidence-based knowledge into everyday care. Only 14% of findings from medical research translates into practice in 17 years [26]. Innovative health care research needs to take into account how research results should be implemented in practice. Such planning impacts the design of research. If the gap between the research setting and the practice is too wide, the results are unlikely to be adopted by practice. Carrying out a randomized controlled trial (RCT) requires extensive resources and rigorous research. There is a need for innovative alternative methods to evaluate complex interventions, such as those developed and evaluated within the U-CARE. The slow implementation rate has resulted in a call for more pragmatic and client-centered research [27,28]. Intellectual property (IP) rights issues and business models affect the implementation of results, and policymaking is a critical factor. A challenge for policymakers is to establish regulations that promote innovation, while still maintaining the public’s trust [29].

Innovation capability may affect research funding, thus this capability is crucial for the survival of the research environment. If research results do not affect health care practice, their value is questionable. Following the innovation ideal, new treatments that prove effective should be implemented into practice.

#### Stakeholders

Innovation is desired by research funders, no matter if funding is commercial, governmental, or comes from nonprofit organizations. Innovation is important both for public health and industrial growth. In addition, in many cases researchers desire that their results be implemented due to commercial or “altruistic” reasons.

#### Managerial Implications

A research environment aiming at innovation needs a strategy to handle IP rights, preferably from the inception of a project. Innovation requires collaboration with, for example, software companies, legal experts, and health care providers. The research environment needs to collaborate with experts in related areas such as implementation science, service management, and business administration to develop business models supporting the translation of results into practice.

### Relevance

#### Theoretical Discussion and Definition

Researchers should be able to explain the societal relevance of their work. Applied research is expected to contribute to society, and research legitimacy is demonstrated in terms of practical relevance [5,30]. As stated, "[...] Researchers should describe the context in which the intervention was developed, applied, and evaluated, so that readers can determine the relevance of the results to their own situation" [5]. Relevance is related to innovation and concerns the kind of knowledge we seek to develop, while innovation emphasizes the translation of knowledge from research into practice. The development of psychological interventions is a complex process. The design literature suggests that the understanding of a certain problem unfolds in the design process [30,31]. Understanding the problem and its relevance is a challenging task. It has been proposed that qualitative [5] and interpretive [32] research is well suited to build a solid understanding of a problem domain, and to formulate hypotheses [33,34]. Relevance in research is a highly discussed ideal, for example, it is used to assess applications for funding and in the peer-review process. Relevance is thus a critical success factor for a research environment.

#### Stakeholders

Stakeholders related to the relevance ideal are similar to those related to innovation. To promote relevance, there is a need to center the design process on health care practice, and include those who will receive the interventions, for example, the patients, the significant others, and those who will provide them, for example, the health care specialists. Relevance builds on an understanding of societal needs. Such needs are periodically investigated and reported by government agencies and the European Union, important stakeholders with regard to relevance.

#### Managerial Implications

Research should proactively adopt a stakeholder-centric design process, including a broad range of stakeholders. The experiences from U-CARE support an iterative approach to development research, interventions, and software. In addition, relevance highlights the need for a continuous monitoring of knowledge gaps and improvement opportunities in the health sector. The research environment needs to engage in intelligence work to understand societal needs in order to maintain and improve the relevance of research.

### Rigor

#### Theoretical Discussion and Definition

Rigor concerns the effective use of knowledge, including both the theoretical foundations and the research methodology throughout the research process [30]. Rigor thus encompasses both the manner in which the researcher selects the appropriate techniques for design and evaluation, and the manner in which the proposed theoretical contributions are justified.

In research on online interventions, it has been argued that trials should lead to an increased understanding of the processes and mechanisms that make treatment effective [5]. Rigor concerns: (1) the way that research methods are enacted, (2) the way that interventions build on existing theory, and (3) that research makes a theoretical contribution. A meta-analysis has shown that the more extensive use of theory in intervention design has a positive impact on effect sizes [35]. Theoretical contributions may include knowledge about processes and mechanisms that make interventions successful, and research methodology, such as novel data collection methods. Researchers in eHealth face new challenges as well as opportunities to collect and analyze data, for example, through the logging of patient behaviors, for example, in forum, chat conversations, and when completing questionnaires [36].

#### Stakeholders

Rigor is important for researchers who aim for high impact publications. It is equally important for journals to maintain and improve their credibility in the academic community. Arguably, publication in high impact outlets strengthens the research environment, and its capability to have an impact on health care practice. Thus, rigor plays an important role as a foundation for research and its meaning for health care practice.

#### Managerial Implications

Senior researchers play an important role in promoting rigor in the research environment. Knowledge management strategies, including formal routines and informal discussions, need to be applied to support all coworkers to continually reflect on the three aspects of rigor outlined above. The elaborate reuse of software and interventions enhances rigor by providing new projects with well tested practices.

### Sustainability

#### Theoretical Discussion and Definition

Something is sustainable if it ‘‘...meets the needs of the present without compromising the ability of future generations to meet their own needs” [37]. Sustainability has been more precisely defined as social, economic, and environmental sustainability [38]. While environmental sustainability has been in focus in IT research (eg, server energy consumption), there is less research on economic and social sustainability [38]. Economic sustainability pertains to how actions contribute to long-term societal development. The contemporary discourse on openness, for example, open source, open content, open data, open standards, and open access publications, relates to economic sustainability. Openness may be beneficial, but it requires new skills and competencies, for example, with respect to legislation and IP rights issues. Social sustainability relates to ethical implications of research, for example, health care should be equally offered to all humans. Social sustainability thus includes the digital divide and access to health care everywhere. As explained by Eysenbach [39], “The digital divide currently runs between rural versus urban populations, rich versus poor, young versus old, male versus female people, and between neglected/rare versus common diseases”. Social sustainability leads to the normative implication that research should take into account the equity implications of new findings.

#### Stakeholders

The European Union Horizon 2020 program, along with some other funding agencies, reinforces sustainability ideals. Social sustainability—promoting equal health care for all—concerns all citizens. However, commercial and public organizations are affected by the trend toward openness, and it’s meaning for organizing and making profits on a market.

#### Managerial Implications

Sustainability adds complexity to organizing research, since it enhances the need for competences in social and ethical issues, IP rights, and the design of technology that benefits society outside the scope of the ongoing trials.

## Results

### The Five Ideals

During 2010-2014, the following five ideals repeatedly occurred while planning and executing research. These ideals constitute a comprehensive rather than complete list: (1) accountability, (2) innovation, (3) relevance, (4) rigor, and (5) sustainability. The challenges regarding each issue of research have been reviewed for their impact on the U-CARE environment.

### Impact on the Uppsala University Psychosocial Care Environment

Accountability was a challenging aspect in the setup of the U-CARE environment and had an impact on how work was organized, and how interventions and software were designed. As psychological treatment is provided within the original trials, Uppsala University (hosting the U-CARE program) registered as a caregiver. A health care organization was set up within the research environment. These organizational implications of accountability were not anticipated initially. The legal and ethical aspects of the management of participant data were continuous concerns radically affecting the software design. Some examples include the way participant data is logged and accessed, the use of double authentication for participants, role-based privileges to access information, and organizational and technical solutions to protect the information. Electronic health record legislation adds further complexity, and health records are at this point managed manually. While accountability issues need to be addressed in the design process, they also need to be aligned with research goals, development, and evaluation of interventions etc.

In order to promote innovation, the U-CARE environment strives toward open sourcing of the software and the interventions. Other research groups may utilize software and interventions developed within U-CARE. Interventions are released under a Creative Commons license, allowing anyone to use them for noncommercial purposes. Research groups associated with U-CARE contribute with new interventions for future reuse. The licensing of software is not yet determined, but the intention is to make it open source. Swedish legislation provides researchers with the IP rights of their innovations and results. Licensing thus becomes subject to a negotiation between contributing researchers. The challenge is to agree about licensing in a way that supports an effective implementation of the software and the interventions, while at the same time resonating with the interests of researchers who are legible IP rights holders. The issue of “effective implementation” cannot be solved without an understanding of stakeholders’, such as IT companies and caregiving organizations having incentives to use and further develop research deliverables. At this point, the U-CARE research environment operates as a service provider. The software is hosted at Uppsala University, providing associated researchers with the opportunity to conduct their studies via the eHealth software. It is a temporary solution, since the university should focus on research, rather than service provisions like IT hosting, software development, and IT support. The university hosts a unit to support innovation, which is periodically consulted by the researchers.

The relevance of the research to the patients is important. Initially, the work was centered on developing interventions and flexible software. In the first feasibility study, directed to adolescents with cancer, the software and the intervention, for example, a self-help program consisting of cognitive behavioral therapy, information, and interactive support, was not received well. Recruitment was difficult and retention was low. In order to improve recruitment, participation, and retention, a group of adolescents with lived experience of cancer was involved in research activities. Patient representatives were also involved in the development of self-help programs for adults with cancer, and adults having had a myocardial infarct. The group of adolescents with lived experience of cancer provided important feedback on various issues, for example, the content of the self-help program, the software user interface, the inclusion criteria, and inclusion procedures. Ideally patient representatives should have been involved in all U-CARE activities from the very start. In the multi-disciplinary setting, there is also exploratory research. As an example, information systems researchers identified relevant research questions during the design process, such as management and design issues concerning privacy and accountability in eHealth.

The U-CARE environment emphasized methodological rigor from the very start. Theoretical foundations have been discussed extensively, but an emphasis has been placed on methodological rigor. However, in response to internal discussions and input from the scientific advisory board, the environment has increasingly paid attention to theoretical foundations and potential theoretical contributions. There are several arenas within U-CARE where methodology and theory is discussed; for example, it is discussed at research seminars, and study coordination group meetings, with the purpose of improving rigor. Rigor is also tightly connected to software design. Well recognized and extensively used instruments are available for reuse in the software, as well as features to improve adherence, for example, rule-based email reminders to participants. In addition, the software has been equipped with extensive, theory-based, logging functionality to improve post hoc scrutiny of patient behaviors. It is believed that such logging is important for rigorous development of theory and for accountability purposes. In essence, rigor is implemented in the organizational routines and in the software to support research.

The aspect of “openness” in U-CARE has been illustrated in the presentation of the innovation ideal. Open sourcing, however, is also a matter of sustainability. In relation to the digital divide, it is clear that eHealth interventions target only part of the population. In the Swedish context, interventions such as those developed and evaluated within U-CARE would make psychological treatment and psychosocial care available for larger groups than they are available for today. First, the online mode of treatment expands the geographical reach of support. Second, at least in the Swedish context, the U-CARE online treatment protocols facilitate support to a group of patients not regularly offered any support today. However, while overall access to psychosocial support and psychological interventions in society is improved, the new form of support may add to the digital divide, due to the dependency on technical equipment and proficiency in using IT. Even though sustainability has affected the work so far, we believe that the research environment would benefit from a more systematic approach to address economic and social sustainability.

## Discussion

### Our Research

Some research has emphasized the operational aspects of online intervention research, for example, the guidelines for conducting online trials [8], and the methods to develop and evaluate complex interventions [5]. Our work contributes to the literature through the focus on research practice at a managerial level. The results, originating from a context of online psychosocial treatment and support, should be seen as relevant for the management of a research environment that conducts research where patients are given care via the Internet. Our approach is thus likely to be useful in other research environments that conduct online trials where patients interact with caregivers.

### A Tentative Set of Ideals for eHealth Research

We suggest that a research environment benefits from systematically scrutinizing ideals that govern research, and their origin in terms of stakeholders. We have identified five ideals and addressed them in a novel way by discussing their implications for managing a research environment. A specific ideal, accountability, originates from the ethics of privacy, and the need to hold people accountable of their actions in case of information misuse or maltreatment. Another two of the discussed ideals, relevance and rigor, are established scientific ideals. Health research is concerned with ethics and the long-term effects of new policies and practices. These concerns are manifested in the ideals sustainability and innovation. Depending on the research context, the ideals may be more or less important to factor into the research design. Given our approach to identify ideals based on a single research environment, it should also be emphasized that a different research context might be influenced by other ideals than those included here. Research management needs to incorporate mechanisms into the environment to continually address ideals, for example, integrating discussions about the proposed ideals into research planning, execution, and reporting.

The result emphasizes the need for different skills, which fuels the argument that an eHealth research environment benefits from a multi-disciplinary collaboration. We have pointed out skills that are of importance to meet each ideal noted in the paper, and the challenges associated with each of these ideals. The need for multiple skills—as well as reuse of knowledge and technology—underscores the complexity of eHealth research. We argue that strong, sustainable, multi-disciplinary research environments are required in order to conduct eHealth research that appropriately addresses the complexity that follows from the five ideals. We have given practical examples of the implications of these ideals by giving examples of the impact of the five identified ideals in the U-CARE research environment.

Finally, ideals are sometimes conflicting. Within the U-CARE program, there is an ongoing discussion about whether open sourcing is an effective way to disseminate results into practice. A commercialization of software and interventions developed within U-CARE might be considered less sustainable, even though commercialization may promote innovation. In addition, research tends to require a trade-off between relevance and rigor. Our goal here is not to provide prescriptions for such decisions; we merely suggest an approach to systematically scrutinize ideals. Such scrutiny informs decisions, and contributes to well reflected multi-disciplinary eHealth research.

## Abbreviations

IP: intellectual property
IT: information technology
RCT: randomized controlled trial
U-CARE: Uppsala University Psychosocial Care Programme
